# Premature Professionalisation or Early Engagement? Examining Practise in Football Player Pathways

**DOI:** 10.3389/fspor.2021.660167

**Published:** 2021-06-07

**Authors:** Liam Sweeney, Dan Horan, Áine MacNamara

**Affiliations:** ^1^Faculty of Science and Health, School of Health and Human Performance, Dublin City University, Dublin, Ireland; ^2^High Performance Department, Football Association of Ireland, Dublin, Ireland

**Keywords:** early engagement, premature professionalisation, youth football, talent pathway, player development, specialisation

## Abstract

There is a growing debate, both in the academic and sporting worlds, about the most appropriate pathway for high potential young players in sport. In this regard, there has been a considerable focus on the age of selection into structured talent development pathways and the nature of the experience once players have been recruited. Given the economic and reputational currency associated with developing professional footballers in particular, it is unsurprising that professional football clubs continue to invest significant financial resources into their academy structures. Understandably, this recruitment policy has attracted substantial attention within the media and research community, with ethical concerns arising surrounding the impact early selection may have on the welfare and the experiences of the young players within the pathway. The aim of this perspective article was to critically consider the research underpinning the early engagement practises of football clubs and the extent to which, and how, the pathway can provide players with the most appropriate starting point for their development. This evidence points to the need to look beyond the prevalent ‘early specialisation vs. diversification’ debate in youth sport towards a consideration of an early engagement perspective that reflects the biopsychosocial influences on talent development and the socio-political environment that influences decisions. We provide practical recommendations focused on the quality of the early engagement experience.

## Introduction

The development of talent in football is big business and, across nations, significant financial resources are invested in identifying and developing talented young players. For example, some football academies in the United Kingdom are now adopting an approach whereby players as young as six are required to attend multiple weekly training sessions, with formal club registration beginning at 9 years of age (Elite Player Performance Plan, 2011; Read et al., 2016). This has led to suggestions of “premature professionalisation” of youth sport to the detriment of the young players involved. Of the 265 million people that regularly play football, only 0.04% play in a professional league (Haugaasen and Jordet, 2012) and even the best performing young players are unlikely to maintain progression and become elite senior players (Sæther, 2018). Furthermore, the complexity of talent identification in sport is compounded by the methods used to select young players into professional academies. Typically, young players are identified and then selected based on subjective analysis by coaches on the factors thought to underpin senior performance (e.g., physical, biological or performance determinants; Williams and Reilly, 2000) without due consideration to the non-linear and dynamic nature of the pathway and the non-stable nature of these factors (Abbott and Collins, 2004). Indeed, the importance of a biopsychosocial approach to talent development has been stressed (e.g., Bailey et al., 2010), and football clubs must consider their role in the biopsychosocial development of players. Reflecting this, the purpose of this perspective article was to critically consider early engagement practises in football and the extent to which, and how, the pathway can provide players with the most appropriate starting point for their development. Framed within the socio-political context of the Irish-UK footballing landscape, the discussions within this perspective article are delimited to male football.

### The Socio-Political Realities of Modern Football

In addition to the complexity of predicting talent at a young age, and reflective of the biopsychosocial approach described earlier, the cultural and societal influences within football must be addressed to account for the complex system that young players experience, and the cultural milieu generated by the sport, context, and even gender. Scouts and coaches base their selection on the extent to which a young player possesses the skills and ability to compete in a specific cultural context or philosophy (Sarmento et al., 2018). The competitive landscape in football also makes talent identification and selection a strategic and tactical decision (Relvas et al., 2010) and there are certainly systemic drivers that force clubs to select young players. Given the cost of buying senior players and the competitive marketplace, it makes sense that professional academies contract large numbers of players at a young age. For example, the player may turn into the next superstar and, for a relatively small outlay, the club will benefit both on (performance) and off (financially) the pitch. Reflecting football's competitive nature, identifying potentially talented players and selecting them at a relatively young age means that they are not available to another rival club. Given the finite coaching resources, money, facilities, and exposure to competition available to all clubs, decisions of who and when to select and deselect on the pathway are inevitable. However, the socio-political landscape of modern-day football is evolving, and clubs must be systematic, careful, and deliberate in designing player development policies. Understandably, there is growing concern about the influence of academy experiences on young players, especially on those released at various, and often early, points on the pathway (Relvas et al., 2010; Mitchell et al., 2020). Significant academic (Brown and Potrac, 2009) and anecdotal (Calvin, 2018) attention has been paid to the deselection experiences of young footballers, with feelings of anxiety, fear, humiliation, and depression experienced by some young players following deselection. Reflecting the professionalisation of football academies, which have also been referred to as “football factories” (Green, 2009, p.7), some football clubs have been accused of viewing young players as commodities and performing bodies that are disposed of once it is deemed that they do not have the necessary qualities and attributes to succeed at the senior level (Brown and Potrac, 2009). Against this basis, there have been calls to reconsider the pathway experience of aspiring footballers and consider ethical issues and the impact of selection and deselection issues within these environments. There is a growing literature base that emphasises the influences of biopsychosocial factors on talent development with recommendations to delay talent identification (i.e., the age of identification) until later in the pathway, to widening talent development opportunities, and expose young athletes to a range of diverse activities across youth sport (Till and Baker, 2020). It may also be that the distinction between early selection and early specialisation, in football academies for example, is less understood and in reality, the context is more complex than the “diversification is good, specialisation is bad” argument that is often cited (cf. Baker et al., 2020).

## Early Specialisation or Early Focus?

Although early sport specialisation has become a popular research area, a universally agreed definition does not currently exist (Jayanthi et al., 2020; Mosher et al., 2020). Initially, Jayanthi et al. (2013) defined early specialisation as “year-round intensive training in a single sport at the exclusion of all other sports.” Jayanthi et al. (2015) later introduced an early specialisation scale, whereby an athlete could be deemed low, medium, or highly specialised based upon three criteria. The authors proposed that the degree of specialisation was influenced by whether the athlete (a) participated in year-round intensive training (more than 8 months per annum), (b) selected one main sport, and (c) quit all other sports to focus on their main sport. Reflecting inconsistencies in terminology throughout the talent development literature (cf. Dohme et al., 2017) a lack of clarity remains, and, in this case, the scale was subsequently questioned because it failed to include all elements that affect an early specialisation pathway, such as training volume and intensity, the type of sport (i.e., individual or team-sport), or the child's autonomy in training (Jayanthi et al., 2020).

Despite the lack of a consensus statement on what constitutes early specialisation (Mosher et al., 2020), and perhaps in response to the structure of competitive youth sport, many researchers and practitioners propose early diversification rather than specialisation as the most appropriate foundation for sporting success (i.e., Bridge and Toms, 2012; DiFiori et al., 2014; Myer et al., 2015; LaPrade et al., 2016; Read et al., 2016; Wilhelm et al., 2017; Güllich et al., 2020). The International Olympic Committee published a statement (see Bergeron et al., 2015) citing generalised concerns associated with youth athletic talent development, including an increased risk of overtraining, burnout and injury. The committee recommended an early diversity of athletic exposure between and within sports, despite acknowledging the need for more definitive evidence. Although there is significant research attesting to the benefits of a diversified early engagement in sport (i.e., Jayanthi et al., 2013; Côté and Vierimaa, 2014; DiFiori et al., 2014), much of the suggestions on early specialisation have been guided by research that is retrospective in design and lacking specificity to football (i.e., Güllich and Emrich, 2006; Law et al., 2007; Wall and Côté, 2007; Fraser-Thomas et al., 2008; Moesch et al., 2011). The latter point is important, as an evidence-based, sport-specific early specialisation definition is needed before strategies for optimal youth participation, injury prevention, and long-term health and performance can be prescribed (Jayanthi et al., 2020).

Some research has attempted to address the risks associated with youth participation in high-level football. A systematic review by Jones et al. (2019) concluded that high-level youth players have a high probability of sustaining a time-loss injury and, consequently, lose large portions of their seasonal development. However, no research within the review included a matched comparator group of diversified sports players tracked prospectively to compare training, match and overall injury incidence rates. In fact, research by Frome et al. (2019) found that specialised high-level youth footballers were less likely to report any previous sports-related injury than non-specialised athletes. Zibung and Conzelmann (2013) and Sieghartsleitner et al. (2018) also reported that to succeed within the Swiss national football system, a vast amount of domain-specific learning activities within early sport participation is recommended. The paucity and low quality of research to date was highlighted in a recent systematic review of youth sports specialisation and musculoskeletal injury (Fabricant et al., 2016). The review included only three appropriate studies, two retrospective studies and one case-control study. Consequently, there is a need for comparative and prospective research to clarify the relationship between youth sports specialisation and musculoskeletal injury (Fabricant et al., 2016).

Although junior success does not necessarily lead to senior success in football (Collins et al., 2016; Taylor and Collins, 2019), there is evidence that early and prolonged engagement in sport-specific activities is related to senior performance. For example, hours spent in football-specific team practise at an early age is associated with expert levels of achievement in English (Ford et al., 2009; Ford and Williams, 2012; Roca et al., 2012), Swiss (Zibung and Conzelmann, 2013; Sieghartsleitner et al., 2018), and Norwegian (Haugaasen et al., 2014) footballers. Although early diversification can be a pathway to elite performance (Coutinho et al., 2016), a diversified early experience has not been shown to be a significant influence on the attainment of expertise in football (Ward et al., 2007; Ford et al., 2009; Ford and Williams, 2012). In fact, the hours accumulated in football-specific play and practise during childhood and youth is a strong predictor for perceptual-cognitive expertise in football (Roca et al., 2012). Methodological limitations (i.e., retrospective study designs, limited to specific cultural contexts) in this research have to be acknowledged, and there is a need for longitudinal and prospective research that examines the microstructure of the different football activities that support development to better inform the design of early experiences in the football pathway (Coutinho et al., 2016; Davids et al., 2017). However, given the socio-political nature of modern-day professional football, and the systemic drivers influencing academy practises mentioned earlier, it is very unlikely that such a prospective, longitudinal and comparative experiment that could provide meaningful insight into the specialisation vs. diversification debate in a football-specific context could ever take place.

## Optimising Early Engagement in a Specialised Pathway

Zibung and Conzelmann (2013) suggest that football requires large quantities of football-specific learning activities during childhood to achieve high footballing performance levels at the age of peak performance. At some stage, a high potential player must prioritise football to maximise his development and fulfil his potential. Baker et al. (2020) highlight the lack of evidence to identify the appropriate time for young athletes to prioritise their chosen sport in an attempt to fulfil their potential. Despite this, Hendry and Hodges (2018) noted that senior professional footballers report that they started playing football early in childhood and, although they did not specialise exclusively in football during childhood, they devoted the majority of their time to it. An analysis from multiple football nations identified that elite players started their participation in an elite football academy at 11–12 years of age (cf. Ford et al., 2012), which is in contrast to current UK football academy practises which formally begin at age nine (Richardson et al., 2004; Read et al., 2016). As there is a lack of empirical evidence to support early childhood recruitment practises, and given that research appears to favour later selection at around age 11–12, it could be suggested that the age at which academies recruit players should be delayed until early adolescence. Côté's Developmental Model of Sport Participation suggests that at the end of primary school (around age 13), children should have the opportunity to either specialise in their favourite sport or to continue in sport at a recreational level (Côté and Vierimaa, 2014). As this investment begins, support must be provided to manage the diverse sporting commitments of young players. This presents an interesting opportunity within a football academy where a young player is often required to commit to 2–4 days of training/match-play per week (Richardson et al., 2004; Mitchell et al., 2020). Given that children aged 5–17 are recommended to engage in 60 min of moderate to vigorous physical activity daily (World Health Organization Physical Activity Recomendations, 2020), an academy player could be encouraged to engage in a range of activities outside the academy programme. For some, this might allow the opportunity to participate in a different sport or activity; for others, it might provide them with the opportunity to play football in a different setting, such as in school or recreationally that would meet their psychosocial needs (Bailey et al., 2010). Given the physical activity recommendations and the current commitment required by young academy players, if managed appropriately, there would appear to be ample time to engage in age, stage, and developmentally appropriate activities in addition to the structured academy programme. Of course, as intensity and physical demands increase, training loads should be monitored to maximise athletic development and minimise the risk of overtraining and injury, especially during rapid growth periods (Wrigley et al., 2012; Jones et al., 2019; Materne et al., 2020).

A key aim of the talent development process is to provide youth athletes with a suitable learning environment to accelerate or realise their potential (Till and Baker, 2020). Kelly et al. (2020) suggested that moving youth footballers into an advanced learning environment may be associated with positive performance outcomes for high potential players. A similar approach is taken in academia, whereby teachers often move high potential students into more advanced educational settings to provide greater learning opportunities (Kelly et al., 2020). Güllich et al. (2017) and Güllich (2019) reported that higher amounts of football-specific free play and structured practise in other sports during childhood, rather than larger quantities of coach-led football practise, differentiated German players at the highest professional standard. However, both studies' findings are restricted by methodological limitations, including retrospective recall bias, cultural limitations, and neither study recorded the “quality” of practise and free play. Contrastingly, a plethora of research (with similar retrospective recall bias limitations) exists to suggest that large amounts of football-specific practise and unstructured free play during childhood contributes to the development of expert performance (Ford et al., 2009, 2012; Zibung and Conzelmann, 2013; Sieghartsleitner et al., 2018). Therefore, this suggests that a developmental pathway should be structured to provide large amounts of football-specific learning activities, but delivered in a broad, diverse, and developmentally appropriate format (Sieghartsleitner et al., 2018), including as examples, coach-led and peer-led practises, peer-led and self-led unstructured free-play, and skill development.

### Bio-Banding: An Example of a Player Development Intervention

Another important consideration when selecting/deselecting players before puberty is the large variation in biological maturation among players of the same chronological age. Children of the same chronological age vary substantially in status (state of maturation) and timing (chronological age at which specific maturation events occur) of maturity (Cumming S. P. et al., 2018). Thus, a youth player competing for selection into a talent pathway may be competing against a player who is biologically advantaged by 6 years (Borms, 1986), despite both players being the same chronological age. Given that early maturation brings physical advantages (such as greater physical size, lean muscle mass, speed, power, and strength; Hill et al., 2020), it is unsurprising that a selection (Cumming S. P. et al., 2018), and performance bias (Hill et al., 2020; Parr et al., 2020) exists in favour of early maturing players.

Categorising youth players based upon biological maturity attributes rather than chronological age is an alternative solution through a process termed “bio-banding” (Cumming et al., 2017). Research into bio-banding in English Premier League academy football has been favourably received by stakeholders (Cumming S. et al., 2018; Reeves et al., 2018). Similarly, “playing up” high potential youth players with chronologically older peers has been suggested to facilitate more appropriate levels of challenge and individual development (Kelly et al., 2020). However, as early biological maturation does not encompass cognitive, emotional, or social development (Cumming S. P. et al., 2018), there is the possibility that categorising players based upon biological maturity alone without considering key psychological developmental influences may be disadvantageous. The influence of biological maturation within youth football is complex and appears to have a greater influence on talent development than the relative age effect (Parr et al., 2020). This is reflected in professional football academy practises, whereby routine monitoring of biological maturity and training load is considered a priority (Salter et al., 2021). More research is needed to conclusively understand performance variations associated with relative age and biological maturity in youth footballers.

### The Dynamic and Non-linear Pathway Experience

It is also crucial to recognise how changes in broader society and talent pathways influence the developmental activities of young players. Research exploring the developmental histories of elite and non-elite athletes (e.g., Baker et al., 2003; Côté et al., 2005; Gulbin et al., 2010), as well as more anecdotal descriptions of free-play, diversification and development (as evidenced in the development of elite Brazilian footballers; Ford et al., 2012), is purported to offer clues to best practise, generating “evidence-based” practises which can be adopted and applied by sports and organisations in the pursuit of excellence. However, western society has changed, and children are unable to acquire the same amount of outdoor free-play than that of previous generations (Solomon-Moore et al., 2018). This descriptive focus also fails to appreciate that talent development is a biopsychosocial issue and optimum solutions will be contextualised based on the interaction between physical and mechanical attributes (the “bio”), psycho-behavioural characteristics (the “psycho”), and the sociocultural environment/milieu in which the individual exists (the “social”) (Collins and MacNamara, 2019). The onus is on sport organisations to critically evaluate the worth and validity of a particular approach (e.g., diversification, specialisation, or early engagement) to provide the most appropriate development experiences in particular contexts. In football, for example, this may be the provision of structures and experiences that allow high potential players to experience sufficient quantities of both football practise, and developmentally appropriate activities *and* unstructured free play within their developmental pathway. Simply, in sports like football where there is less evidence of the discriminatory power of broader activity (Haugaasen et al., 2014), early selection into an academy setting may not have negative consequences as long as high potential players are provided with an enrichment programme of other activities; a focus on early engagement rather than early specialisation.

The talent development process is non-linear (Collins and MacNamara, 2012) and the complex transition from youth to professional football (Larsen et al., 2014) could be cited as an argument against early selection. Players should be able to transition in and out of the pathway across multiple time points as they progress, although the reality appears to be more complex, and the number of athletes reaching elite levels is constrained by the numbers of professional players a system can maintain. Simply, deselection from football academies is inevitable for the vast majority of players at some stage, and if poorly managed, it can have negative emotional and psychological impacts on young players (Brown and Potrac, 2009). Athletes have also reported questioning their identity, their ability as an athlete, and the role of sport in their lives following deselection (Neeley et al., 2018; Mitchell et al., 2021). Football clubs have a moral and ethical responsibility to focus on the welfare of all players under their care, whether they are selected to progress further or deselected.

However, the pathway experience for deselected young athletes can also be a positive one. Williams and MacNamara (2020) found that high-potential young athletes who were deselected reported that the experience of the talent pathway provided the foundations for future success in other sports, careers, or education opportunities. The talent pathway can provide an environment that develops valuable constructs, (i.e., professionalism and positive performance behaviours), psychobehavioural skills (i.e., social awareness and effective communication), and personal responsibility (i.e., self-motivation and personal drive/desire) which can crossover to alternative domains outside of sport and prove advantageous (Williams and MacNamara, 2020). Similarly, Neeley et al. (2018) identified that deselection from the talent pathway can be accompanied by subsequent personal growth experiences. The authors noted that, despite not progressing, deselected athletes experienced an enhanced sense of personal strength, developed closer social relationships, and recognised new and alternative opportunities. Therefore, it is possible that if structured appropriately, players can have many positive experiences during their time in football academies leading to the development of multiple skills and behaviours that are transferable to many other parts of their lives (Williams and MacNamara, 2020).

It is unlikely that football clubs will cease to select young players at a young age and in fact, the research suggests that early specialisation issues may be less influential in sports like football which requires higher skill and variability than in athletics, for example (Paul et al., 2016). The focus should be on the quality of the experience offered to high potential young players in order to support their development both on and off the pitch (Strachan et al., 2011; Kelly et al., 2020; Williams and MacNamara, 2020).

## Discussion

Despite the non-linear nature of talent development, there is a lack of research investigating those who do not make it to the highest level, perhaps leading to survivorship bias within the literature (Taylor and Collins, 2019). The need for more research is evident, and issues of biological, neurological, and social readiness have rarely been considered (Baker et al., 2020). We must consider the viewpoints of the coaches, and critically, the high potential young athletes themselves regarding developmental strategies, since their voices have been largely absent from the discussion to date (Baker et al., 2020). When examining the development of young footballers, it is essential to embrace an interdisciplinary approach (rather than a monodisciplinary one) and adopt a longitudinal design (rather than a cross-sectional one) in the hope of better understanding the prospective value of various influences, and how these may gradually oscillate as players age and are exposed to systematic training (Williams et al., 2020).

Selection and deselection are an inevitable part of any pathway and care is needed in both the decision-making process and the language used to describe these decisions. We offer some practical examples.

- A player's deselection from the highest playing level of the pathway should not be deemed early elimination, but rather as an opportunity for players to be directed to a football environment that is better suited to their needs at *that* stage of their development. A key aim of the talent development process is to provide players with the best possible environment to support their trajectory and development. Nurturing players along a dynamic pathway in environments best suited to their individual needs at particular points of childhood and adolescence requires a system that facilitates multiple entry, exit and re-entry points. This type of coherent development system may seem aspirational given the socio-political context of modern-day football. Nonetheless, the large gap between research theory and sporting practise that exists (cf. Pankhurst and Collins, 2013) must be addressed and it is the responsibility of those working within the football industry at every level to ensure that the welfare of children is at the forefront of all decision making. If conditions can be created whereby an academy manager feels secure in the knowledge that producing players is not the sole outcome measure, a positive player development model can be woven into academy culture (Mills et al., 2014). From a “top-down” approach, this may require the refinement of current governance of youth football and increased collaboration and coherence across the youth football landscape. From a “bottom-up” perspective, it points to the importance of quality academy coaching that recognises the complexity and dynamic nature of the developmental process (UK Sport Pathway Coaching Position Statement, 2020).
- Football clubs and systems should look to provide young players with a variety of playing formats (i.e., modified pitch sizes and futsal), coaching styles (i.e., introduce peer-led free play alongside coach-led practise and competition), and competition structures (i.e., various team sizes and match durations) to provide a football-specific learning experience, but delivered in a broad, diverse, and developmentally appropriate format (Sieghartsleitner et al., 2018).
- We must consider the socio-political factors within organisational structures that influence player development. A lack of formal communication between youth and professional environments, even within the same club or broader system, can hinder the coherent progression of young players into the professional environment (Relvas et al., 2010). In practical terms, this means that open and clear lines of communication need to exist between clubs and coaches operating at different levels of the game so that players are supported in their development as they progress from youth to adult football.
- Given that many talented young athletes are derailed from the pathway by a range of psychological influences, it would seem prudent that the academy experience supports the development of the psychological skills and characteristics shown to support development within, and transfer between, sports and domains (MacNamara et al., 2010a,b; Taylor and Collins, 2019). Such practises could be facilitated using the 5C's (commitment, communication, concentration, control and confidence), providing the successful, and crucially, early introduction of interpersonal and psychological skill development into coach, player and peer interactions to facilitate long-term development (cf. Harwood, 2008).
- Football clubs and broader systems must adopt a holistic approach to player development, whereby players are provided with the tools and resources to develop as people and succeed both in and outside of football (cf. Larsen et al., 2020). Coaches should provide an environment whereby the teaching of life skills (i.e., effective communication, exhibiting leadership, taking the initiative) is emphasised and integrated alongside the teaching of sport-specific skills (Bean et al., 2018). This becomes sustainable once coaches are able and encouraged to discuss and practise life skills and skill transfer with their athletes (Camire and Santos, 2019). Under these circumstances, there is a focus on the overall development of the person, rather than just the footballer.
- Parents are a key stakeholder in the talent development environment with essential and individualised roles and responsibilities (Côté, 1999; Pankhurst and Collins, 2013). However, many parents of academy players have reported experiencing insufficient levels of communication, feeling undervalued, being treated with a lack of empathy, and feeling worried and uncertain about their child's welfare and future (Harwood et al., 2010; Clarke and Harwood, 2014). To bridge this gap, academy staff must make a conscious effort to spend time developing relationships with parents in order to provide tailored and continuous support in an environment where parents feel welcomed, valued and respected (Newport et al., 2020). Moreover, clubs must make an effort to educate parents early in relation to the necessary motivation-related knowledge and cognitive-behavioural skills to manage themselves and facilitate their child's development throughout their time in the academy (Harwood et al., 2010; Newport et al., 2020). This education may be provided in the form of parent inductions upon entrance into the academy, followed by regular educational workshops and open and transparent discussions between parents, coaches and academy management staff.
- Professional football clubs must make an effort to collaborate with local grassroots clubs and schools, thereby emphasising vertical coherence within the sport (Webb et al., 2016). This may include opportunities for players from grassroots clubs and schools to train with the academies of professional partner teams, regular school holiday camps hosted at partner club locations, and regular fixtures between professional and grassroots clubs.
- Finally, the “rocky road to the top” (Collins and MacNamara, 2012) must be one that is systematic, well-planned, and coherent. The pathway must enable players' transition in and across challenging environments in a structured and supportive manner (Webb et al., 2016). This nested and nurtured approach to player development, with appropriate, well-planned and periodised developmental challenges and experiences (Collins et al., 2016), only becomes achievable when all of the key stakeholders in the game collaborate for the best interests of players.

## Data Availability Statement

The original contributions presented in the study are included in the article/supplementary material, further inquiries can be directed to the corresponding author.

## Author Contributions

All authors listed have made a substantial, direct and intellectual contribution to the work, and approved it for publication.

## Conflict of Interest

The authors declare that the research was conducted in the absence of any commercial or financial relationships that could be construed as a potential conflict of interest.
